# EFFECTS OF SOCIAL SUPPORT NETWORK ON AMERICAN OLDER ADULTS’ MEMORY FUNCTIONS

**DOI:** 10.1093/geroni/igz038.3383

**Published:** 2019-11-08

**Authors:** Ethan Siu Leung Cheung, Kedong Ding

**Affiliations:** 1 Columbia University, New York City, New York, United States; 2 Community Counseling & Mediation NYC, New York, New York, United States

## Abstract

Background: Previous studies have found older adults’ cognitive functions are strongly associated with their social networks, including memory. Yet, few studies have explored the influences of specific social network members, such as siblings and children. Further, little studies examined the impact of the size of older adults’ social networks. Hence, this study aimed to investigate how older adults’ relationships with their spouses, siblings, and children, as well as the size of their social networks, affect American older adults’ memory functions. Methods: Using the 2018 data from NHATS, 5547 samples were included. We adopted a multiple logistic regression model to test the impact of social support network sizes, and how associations of social support networks varied between spouses, siblings, and children. All models were calibrated for age, gender, education, income, and race/ethnicity. Results: Analysis showed that higher socioeconomic status (more education and without Medicaid), being female, and younger age were associated with increased odds of having good self-rated memory functions. Older adults with larger social support networks (>=3 individuals) were more likely to have better self-rated memory function (adjusted odds ratio, 1.182, p<0.05), while holding other variables. Having a spouse also increased odds of higher self-rating memory function, in contrast to having children. Conclusion: This study highlighted the importance of having a larger social network size for older adult’s memory function and indicated the necessity of developing intervention programs to expand older adults' social network size, especially for those with lower socioeconomic status.

